# Some American Hospitals

**Published:** 1891-12-12

**Authors:** 


### Some American Hospitals.

By Our Special Commissioner.

### I.—Introductory.

The more one travels the more patent does it become that the efficient administration of the hospitals in every part of the world is dependent upon the same factors everywhere. No one who is familiar with the condition, arrangements, and management of British and American hospitals will dispute that, relatively, the former are much more efficient than the latter. This is due to three principal causes:

Firstly.—In Great Britain there is almost everywhere a wholesome public spirit, and the maintenance of each hospital is largely dependent upon the support of the public, who, giving their money, not unnaturally take a warm interest in the condition of the institution to which they subscribe. In America, on the other hand, public spirit at the present time is almost, if not entirely, wanting. If a man suffers injury from his fellow man, his idea is that he has not the time, nor can he, in reality, afford to recover from the debtor that which is due to him, and so he solaces himself by taking it out of the next man he comes across in the way of business. It is, probably, largely true that the very many Americans regard the men who can pile up dollars most rapidly as the greatest, best, noblest, most powerful, and most admirable examples to be found in their country to-day. It would be a wrong statement to say, what was undoubtedly true thirty years ago, that thieving and lying were principles which a very large number of Americans regarded as justifiable in the way of business. Happily the American people are beginning to see, slowly, no doubt, but we hope surely, that there is something better and more desirable, and altogether greater than the worship of the almighty dollar. Still, an absence of public spirit makes itself felt in the administration of many of the American hospitals, and so, taken as a whole, they are relatively inefficient, as compared with those of Great Britain.

Secondly.—It is difficult in America to command the services of capable administrators for the conduct of these institutions, and, although some excellent examples might be quoted—the Johns Hopkins Hospital, Baltimore; University Hospital, Philadelphia; and the City and General Hospitals, Boston, to wit—where the highest state of efficiency prevails, owing to the fact that able and devoted men have been found to do the work in American hospitals, as a class, even where the buildings are planned upon approved principles, the general administration is far below the mark. This is doubtless due to the fact that many of them are in charge of lay Superintendents, whose appointments are often political, and who, necessarily, are selected for the most part for services rendered to their party rather than for their capacity and experience in hospital management.

Thirdly.—Many of the hospitals are rate-supported, or, what is worse even than that, are under the management and control of the Municipalities, *i.e.*, of the local politicians.

A striking example of this latter class is to be found in the city of San Francisco, than which no city is more admirably placed or circumstanced in regard to climate and surroundings for the treatment of all classes of disease under the most favourable conditions. The chief hospital in San Francisco is constructed upon a plan which is adapted to the size and importance of the institution itself. The method of administration laid down in the Rules and Regulations is based on sound principles, and provides that the administration of the whole of the staff shall be under the control and direction of a resident Medical Superintendent. Yet an inspection of the hospital shows that, despite all these advanatges, it presents a sad aspect of neglect. Everywhere evidence is forthcoming to the trained observer that efficiency, insistence upon the thorough discharge of the duties attaching to each office, and a stern enforcement of rules and regulations, are nowhere to be met with in this establishment. No doubt great responsibility attaches to the Medical Superintendent, but as the rules provide that he cannot dispense with the services of the humblest employé without first obtaining the sanction of his Committee it may easily be understood that although, without doubt, his authority is nominally supreme, in practice his orders are disregarded, and so inefficiency and neglect prevail, we fear, to a large extent.

It is true that examples of nearly every system of administration may be found in America. The methods are somewhat different, but the results are similar. Thus, hospitals are maintained by various charities or religious organizations, and we find Episcopalian hospitals, Presbyterian hospitals, Jewish hospitals, Methodist hospitals, and a host of Roman Catholic Institutions are supported by the respective churches, with the exception of the Roman Catholics, who seem to depend largely upon the support of the general public to supplement any deficiency which may exist between the amount received from patients and the total expenditure of each year.

Unfortunately, the Church administration of Hospitals as represented by American institutions, does not invariably produce efficiency. This is no doubt due to the relatively narrow view which may be taken by a particular church, and of the system of administration which should be enforced.

On the whole, it is fair to say that the hospitals which owe their origin and maintenance to churches are relatively more efficient than those which come directly or indirectly under the control of local authorities in each town. It is, morever, very satisfactory to be able to report that there has been a marked improvement in the quality of the administration of American hospitals, taken as a whole, during the last ten years. When we inspected them in 1882 there was hardly one which was entirely satisfactory. Now several are highly efficient. The improvements in the nursing arrangements especially, and in the provision made for the comfort and skilled instruction of nurses, are as remarkable as they are praiseworthy and commendable.

---

The *Brooklyn Medical Journal* draws attention to a method of resuscitating drowned persons, which a Dr. Harvey, through force of circumstance, was compelled to adopt, and which proved successful. A young man out boating capsized his boat, and was seen by the doctor, who was also out boating; he pulled him in at the bow quite relaxed and unconscious. The doctor then placed him on his back, his shoulders on a thwart, the back of his head resting on the bottom of the boat, arms extended, as in Sylvester's method. As soon as the head was depressed water poured from mouth and nose, and increased in flow when the shoulders were raised still higher than the level of the head. As the flow ceased, the man gasped, and his shoulders were lowered to the thwart again. Sylvester's method of artificial respiration was not necessary, as the respiration gradually improved, the arms being left extended. The patient was then rowed to shore, and in five hours recovered consciousness. Considering how often delay in action causes death, this method had, in this case at any rate, the merit of being able to be carried out in the boat. It would be interesting to hear of any similar experience.

